# Novel Compound Heterozygous Variants of *ETHE1* Causing Ethylmalonic Encephalopathy in a Chinese Patient: A Case Report

**DOI:** 10.3389/fgene.2020.00341

**Published:** 2020-04-17

**Authors:** Xiaohong Chen, Lin Han, Hui Yao

**Affiliations:** ^1^Department of Endocrinology and Metabolism, Wuhan Children’s Hospital, Tongji Medical College, Huazhong University of Science and Technology, Wuhan, China; ^2^Running Gene Inc., Beijing, China

**Keywords:** ethylmalonic encephalopathy, *ETHE1*, elevated ethylmalonic acid, chronic diarrhea, genetic sequencing

## Abstract

Ethylmalonic encephalopathy (EE) is a very rare autosomal recessive metabolic disorder that primarily affects children. Less than one hundred EE patients have been diagnosed worldwide. The clinical manifestations include chronic diarrhea, petechiae, orthostatic acrocyanosis, psychomotor delay and regression, seizures, and hypotonia. The *ETHE1* gene has been shown to be associated with EE, and genetic sequencing provides concrete evidence for diagnosis. To date, only 37 variants of *ETHE1* have been reported as disease-causing in EE patients. We identified two novel *ETHE1* variants, i.e., c.595+1G>T at the canonical splice site and the missense variant c.586G>C (p. D196H), in a 3-year-old Chinese boy with EE. The patient had mild symptoms with only chronic diarrhea. The typical symptoms, including spontaneous petechiae, acrocyanosis, and hypotonia, were all absent. Herein, we report on the clinical, biochemical, and genetic findings of our patient and review the phenotypes and genotypes of all patients with EE caused by *ETHE1* variants with available information. This study supports the early assessment and diagnosis of EE.

## Introduction

Ethylmalonic encephalopathy (EE, OMIM#602473) is a rare, early-onset metabolic disorder of infancy that affects the development of the brain, gastrointestinal tract, and peripheral blood vessels. EE was first identified in 3 unrelated Italian children as a branched-chain acyl-CoA oxidation defect in 1991 (Burlina et al., 1991), but most patients diagnosed with EE were of Mediterranean and Arabic descent (Tiranti et al., 2004). To date, only two EE patients have been reported in China (Li et al., 2014; Zhang et al., 2018). However, EE always leads to death within the first decade of life in the most of children (Walsh et al., 2010).

In 2004, for the first time, recessive variants of *ETHE1* (ethylmalonic encephalopathy protein 1) were identified as the causes of EE (Tiranti et al., 2004). *ETHE1* (chromosome 19q13.32) encodes a mitochondrial sulfur dioxygenase involved in sulfide detoxification. The impairment of sulfur dioxygenase leads to the accumulation of hydrogen sulfide (H_2_S) and its derivatives, and the major clinical manifestations of EE are mediated by hydrogen sulfide accumulation, which damages mucosal cells in the large intestines and vascular endothelial cells and is associated with chronic diarrhea. Hydrogen sulfide is also vasoactive and vasotoxic, which could explain the symptoms of petechiae and orthostatic acrocyanosis in EE (Tiranti et al., 2009). Vasculopathy is also associated with multiple necrotic brain lesions, resulting in hypotonia, psychomotor delay and regression, predominant pyramidal signs, seizures, and eventually global neurological failure (Walsh et al., 2010; Dionisi-Vici et al., 2016). Regarding biochemical examinations, the disorder is characterized by elevated urinary ethylmalonic acid (EMA), methylsuccinic acid (MSA), lactic acid, C4-/C5-acylcarnitine esters and plasma thiosulfate. The toxic levels of hydrogen sulfide are responsible for these biochemical alterations because they inhibit the activities of cytochrome *c* oxidase and short-chain acyl-CoA dehydrogenase, especially in skeletal muscle (Tiranti et al., 2009; Di Meo et al., 2011).

Generally, next-generation sequencing is a good approach used to support the clinical diagnosis of EE. According to the Human Gene Mutation Database professional version (HGMD pro) (Stenson et al., 2003), only 37 variants of *ETHE1* have been identified as disease-causing. Expanding the variant spectrum of *ETHE1* is necessary for the diagnosis of and further research investigating EE. Herein, we report the clinical, biochemical and genetic findings of a 3.5-year-old Chinese boy with EE who carries two novel *ETHE1* variants.

## Methods

This research was approved by the Medical Ethics Committee of Wuhan Children’s Hospital. The patient’s parents were informed and provided written consent for the study and the publication of this report.

### Case Presentation

**FIGURE 1:**
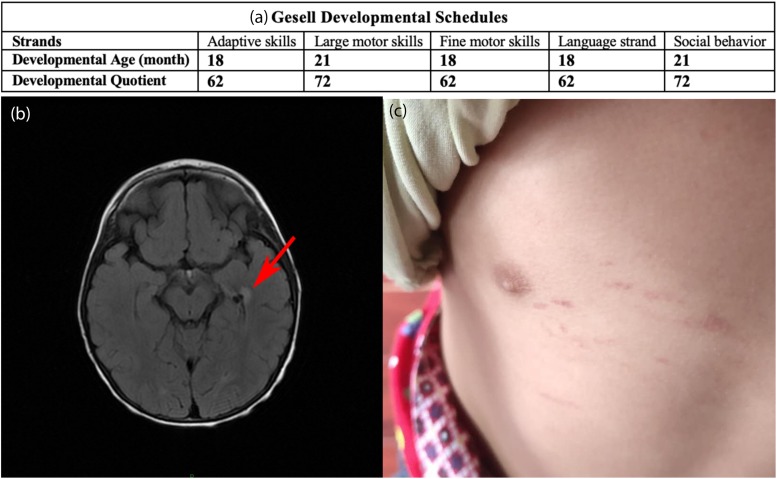
**(a)** Results based on the Gesell Developmental Schedules. Chronological age is 29 months. Developmental quotient<75 indicates developmental delay. **(b)** Brain MRI. The red arrow indicates shadows in the temporal horn of the left lateral ventricle. **(c)** Petechiae on the skin caused by falling appeared on the patient.

The patient, a 2-year-old Chinese boy referred to our hospital in 2018, was born to non-consanguineous parents. When the child was born, he presented with chronic diarrhea (3–6 times per day) which lasted for two years. Four months before the patient was admitted to our department, he was diagnosed with superficial gastritis and colitis at the Gastroenterology Department. Oral probiotic supplements were given to the patient, but he failed to respond to the treatment. Chronic diarrhea remained, but no developmental delay was observed. A urine analysis showed obviously elevated EMA [71.84, 61.89, and 28.96 μmol/L; reference value (r.v.) 0.00–6.20], slightly increased lactic acid (9.05 μmol/L, normal, normal; r.v. 0.00–4.70) and increased isobutyryl glycine (1.85 μmol/L, r.v. 0.00–0.40). After admission to our department, tandem mass spectrometry revealed increased C4-acylcarnitine (0.91 Um, r.v. 0.06–0.50) in the blood. The assessment based on Gesell Developmental Schedules^1^ showed that the developmental age was approximately 18 months and indicated overall developmental delay, especially in adaptive skills, fine motor skills, and language (Figure 1a). Brain MRI (Figure 1b) showed abnormal signal shadows in the temporal horn of the left lateral ventricle, revealing demyelination in the brain. Petechiae on the skin caused by falling appeared on the patient (Figure 1c). However, spontaneous petechiae, acrocyanosis, and hypotonia were absent in the patient. Regarding treatment, L-carnitine, vitamin B1 and vitamin B2 were given, but this treatment was ceased due to due aggravation of diarrhea. *Clostridium butyricum* tablets were given continuously but chronic diarrhea remained. Except for a language delay, the patient did not exhibit any other evident abnormalities during a telephone follow-up. In early 2020, a combination of metronidazole and N-acetylcysteine was given based on previous studies (Viscomi et al., 2010). The patient will be continuously followed up, and liver transplantation will be considered if the disease cannot be controlled.

### Whole-Exome Sequencing

Whole-exome sequencing (WES) was applied to support the diagnosis. Peripheral blood samples were collected from the patient and his parents and sent to Running Gene Inc. (Beijing, China) for WES. DNA samples were extracted and qualified using a DNA Isolation Kit (AU1802, Bioteke) and Qubit dsDNA HS Assay Kit (Q32851, Invitrogen, United States). The qualified DNA samples were fragmented into 200–300 bp fragments and then prepared with a KAPA Library Preparation Kit (KR0453, Kapa Biosystems) according to the manufacturer’s protocol. Hybridization of the prepared libraries to the capture probes was carried out according to the instructions for the IDT and xGen Lockdown^®^ Probes (Integrated DNA Technologies Inc., United States). The captured DNA samples were sequenced on a HiSeq X10 (Illumina, United States). High-quality paired-end reads were aligned to the human reference genome sequence hg19 from the UCSC genome browser (Kent et al., 2002) with the BWA tool (Li and Durbin, 2009). Single-nucleotide variants (SNVs) and insertions and deletions (INDELs) were filtered using GATK (Van der Auwera et al., 2013). All called variants were annotated based on public databases, including the 1000 Genomes Project (Genomes Project et al., 2015), ExAC (Lek et al., 2016), and gnomAD (Karczewski et al., 2019). China National GeneBank (CNGB)^2^ was also used to validate the novelty of the variants in the Chinese population. We analyzed the pathogenicity of the variants mainly based on criteria following the American College of Medical Genetics and Genomics (ACMG) guidelines (Richards et al., 2015). Sanger sequencing was applied to validate the familial segregation of the variants.

## Results

**FIGURE 2:**
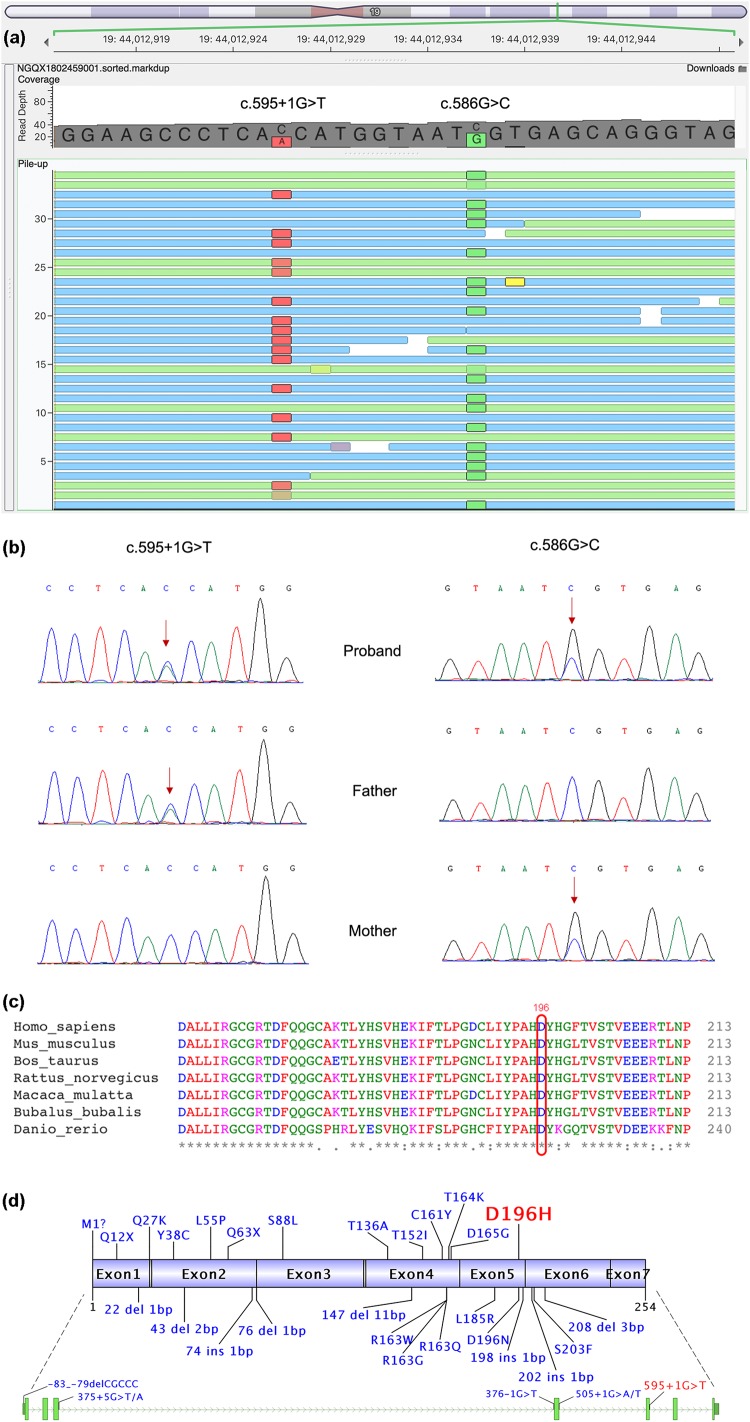
Whole-exome sequencing and Sanger sequencing results. **(a)** Two variants of *ETHE1*, c.586G>C and c.595+1G>T, were identified. **(b)** Sanger sequencing of *ETHE1* performed to verify the variants in the proband. Sanger sequencing confirmed that the c.595+1G>T variant was inherited from the father and that c.586G>C (p. D196H) was of maternal origin. **(c)** Multiple alignment of the ETHE1 protein sequence across several species. Variant site Asp196 is labeled by a red circle. * in the last row indicates “highly conserved.” **(d)** Nineteen missense variants, 8 insertions/deletions, 5 splicing variants and 1 variant in the noncoding region are labeled in blue (two variants leading to p. M1?). Our case shows c.586G>C (p. D196H) in exon 5 and c.595+1G>T at the canonical splicing site (red). The upper panel is the ETHE1 protein sequence (exon n indicates the exon n-encoding region). The bottom panel shows the *ETHE1* gene sequence, including the exons, introns, and noncoding regions.

The patient carried two novel compound heterozygous variants of *ETHE1* (NG_008141.1, NM_014297.5, NP_055112.2) (Figures 2a,b). The variant with a paternal origin, i.e., c.595+1G>T, is a null variant located at the canonical +1 splice site of exon 5 (PVS1) and likely affects splicing, resulting in the loss of protein function. The variant is absent from controls (1000 Genomes, ExAC, gnomAD, and CNGB) (PM2). Multiple computational software programs predicted that the variant probably has deleterious effects on splicing (PP3). Human Splicing Finder v3.1 predicted that the variant may alter the WT donor site, most likely affecting splicing (Desmet et al., 2009). Similarly, the NetGene2 Server^3^ suggested the disappearance of a donor splice site after the alteration. Thus, c.595+1G>T is considered a pathogenic variant. In addition, the maternal missense variant c.586G>C in exon 5 leads to an amino acid change (p.D196H). This recessive variant is absent from controls (PM2) and is in *trans* with the pathogenic variant c.595+1G>T (PM3). This variant is also predicted to be a deleterious variant by several computational software programs (PP3). Mutation Taster, SIFT, Provean and Polyphen-2 predicted it as disease-causing (probability 1.000), damaging (score 0.001<0.05), deleterious (score −6.93 < −2.5) and probably damaging (score 1.000), respectively (Choi, 2012; Choi et al., 2012; Adzhubei et al., 2013; Schwarz et al., 2014). Although c.586G>C is a novel variant, c.586G>A (p. D196N) has been reported in the HDMG pro database as a disease-causing variant at the exact same site (PM5) (Mineri et al., 2008), indicating the pathogenicity of the novel variant of *ETHE1*. In addition, the site of this residue is highly conserved in the *ETHE1* sequence across species (Figure 2c). Therefore, c.586G>C is classified as a likely pathogenic variant.

## Discussion

EE is a devastating recessive mitochondrial disorder with only approximately 100 patients diagnosed worldwide. There is no specific treatment for EE patients, and research concerning disease is quite rare. Currently, developing a targeted therapy for EE is not an economical idea, but it is necessary to improve the processes used for the diagnosis of EE. Compared with physical and biochemical examinations, the sequencing of the responsible gene *ETHE1* is an essential and direct approach used to diagnose patients with EE, although the results still need support from conventional examinations. Genetic sequencing also helps in differential diagnosis between EE and multiple acyl-CoA dehydrogenase deficiency (MADD), which is caused by *SCAD* variants (Zafeiriou et al., 2007).

Only 37 disease-causing variants of *ETHE1* are listed in HGMD pro, including 19 missense variants, 8 insertion and deletion variants, 4 exon deletion variants and 5 splicing variants, including 3 at canonical splicing sites (Figure 2d). Since 9 variants are located in the 4th exon and the 4th exon is involved in all exon deletions, we consider the 4th exon a hot region for disease-causing variants, highlighting the importance of its structural and functional integrity. In addition, in our case, we identified two novel variants, including one missense variant (c.586G>C; p. D196H) and one splicing variant (c.595+1G>T) to enrich the database of *ETHE1* variants.

**FIGURE 3:**
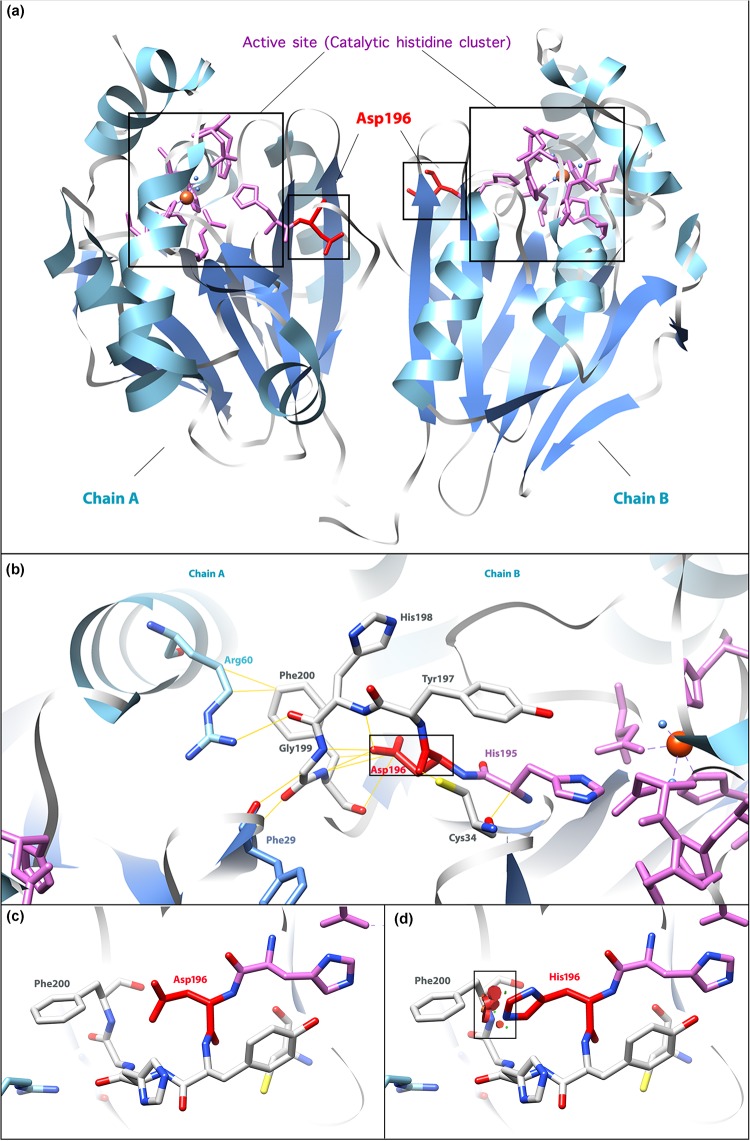
Molecular model of the human ETHE1 protein. **(a)** The active form of the ETHE1 protein is a dimer, including chains A and B. α-helices and β-sheets are labeled in light blue and cornflower blue, respectively. The active site (catalytic histidine cluster) is the iron-binding region. The residue affected in the missense variant, Asp196, is shown in red. **(b)** Interactions between Asp196 and surrounding residues. Yellow lines show interactions between labeled residues in ETHE1. **(c)** Wild-type Asp196 and Phe200. **(d)** Mutant His196 and Phe200. Powerful repulsions between two residues are labeled in the black square. Three-dimensional structures were generated based on the data from Pettinati et al. (2015) by the UCSF Chimera (Pettersen et al., 2004).

In the active form of ETHE1, a mitochondrial sulfur dioxygenase is a dimer of two identical monomers, chain A and chain B (Figure 3a) (Pettinati et al., 2015). ETHE1 contains an αββα metallo-β-lactamase fold, which supports metal binding by catalytic histidine clusters and aspartate (Papetti et al., 2015). The 4th exon encodes a β-sheet as a part of the active site, which explains the significance of its integrity. Asp196 is located near the catalytic histidine cluster (His195) and interacts with several residues (Cys34, Tyr197, His198, Gly199, and Phe200), which might contribute to the maintenance of the monomeric or dimeric structure (Figure 3b). The alteration from Asp196 to His196 generates a powerful repulsion between His196 and Phe200, probably reducing the stability of the EHTE1 protein structure (Figures 3c,d). Unfortunately, this speculation cannot be verified since it is not possible to retrieve biological material from the patient. However, it has been reported that the missense variant c.586G>A (p. D196N) does not influence the expression level of ETHE1 protein; thus, this variant likely affects the catalytic activity of EHTE1 (Mineri et al., 2008). Therefore, it is probable that the novel variant c.586G>C (p. D196H) in our case alters the conformation of the catalytic histidine cluster or the structure of the dimer, thus indirectly interfering with substrate recognition and catalysis.

EE is characterized by chronic diarrhea, petechiae, orthostatic acrocyanosis, hypotonia, pyramidal signs, psychomotor delay and seizures. Biochemically, elevated urinary EMA, MSA, lactic acid and C4-/C5-acylcarnitine ester are signs of EE. However, the clinical manifestations of patients vary depending on the individuals and the variants they carry. The Table 1 reviews the published variants and their corresponding clinical phenotypes and includes the current case. Elevated EMA and C4-acylcarnitine ester are present in all cases (15/15) and are necessary conditions for an EE diagnosis. Psychomotor delay (14/15), chronic diarrhea (10/15), hypotonia (11/15) and elevated serum lactic acid (12/15) are typical features. Half of all patients also experience symptoms, including petechiae (8/15), acrocyanosis (6/15), pyramidal signs (6/15), and seizures (6/15). The clinical synopsis of EE in OMIM^4^ recorded that retinal lesions with tortuous vessels is among the manifestations, but only the present case was associated with abnormal visual evoked potentials (1/15). Moreover, allelic heterogeneity is likely present in our case and case 2. These two patients shared the same locus but different variants and showed relatively mild symptoms compared to that of the other patients, supporting the opinion that the variant on Asp196 is associated with a benign course of EE. Since orthostatic acrocyanosis and the pyramidal signs in case 2 were identified at the age of 5, follow-ups of our patient will be necessary to determine the presence of allelic heterogeneity. Since our patient has already shown petechiae caused by falling at 3 years of age, it is likely that spontaneous petechiae or acrocyanosis will emerge in the near future.

**TABLE 1:** Variants and clinical manifestations of EE patients.

**Case**	**Variant**	**Age at onset**	**Development**	**Chronic diarrhea**	**Petechiae**	**Acrocyanosis**	**Hypotonia**	**Pyramidal sign**	**Seizures**	**Cytochrome c oxidase deficiency**	**EMA**↑	**MSA**↑	**Lactic acid**↑	**C4 – carnitine**↑	**C5 – carnitine**↑	**Eyes**	**Brain MRI**	**Other clinical findings**	**Exitus**	**References**
1	c.586G>C p.D196H and c.595+1G>T splice in ETHE1	2 years 5 m	Global developmental delay	+	+ (only after falling, 3 years)	−	−	−	−	N/A	+	+	+	+	−	Abnormal visual evoked potentials	Abnormal shadow next to the left lateral ventricle	Abnormal auditory evoked potentials in the brainstem	−	Present case
2	Homozygous c.586G>A p.D196N in ETHE1	6 m	Psychomotor delay and regression	+	−	+ (5 years)	+	+ (5 years)	−	58%	+	−	+	+	N/A	−	Bilateral asymmetrical high T2-weighted signal intensity in the globus pallidus, capsula extrema, and amygdala	Vomiting	−	Mineri et al., 2008
3	c.221–222insA p.Y74X and c.491C>A p.T164K in ETHE1	1 week	Psychomotor delay and regression and poor growth	+	+	+	+	+	+	28%	+	+	+	+	+	−	Symmetrical bilateral lesions in the striatum and globus pallidus	−	17 m	Mineri et al., 2008
4	Homologous c.164T>C p.L55P in ETHE1	6 m	Psychomotor delay	−	+	+	+	−	+	30%	+	−	+	+	+	−	Abnormal signal in the white matter with Leigh-like lesions	−	−	Mineri et al., 2008
5	c.487C>T p.R163W and c.455C>T p.T152I in ETHE1	6 m	Psychomotor delay and neurological regression	−	−	−	+	−	+	N/A	+	−	N/A	+	+	−	N/A	−	−	Mineri et al., 2008
6	Homologous del ex.4–7 in EHTE1	1 week	Psychomotor delay and regression	+	+	+	+	−	−	N/A	+	+	+	N/A	N/A	−	Brain atrophy	Upper respiratory infection	−	Mineri et al., 2008
7	c.66delC stop codon in ETHE1 and c.625G>A in SCAD	5 m	Failure to thrive	+	+	−	+	−	−	+	+	+	+	+	+	−	Cortical atrophy (EEG)	Enlarged kidneys and diffuse mesangial sclerosis	−	Merinero et al., 2006
8	c.488G>A p.R163Q and c.375+ 5G>T splice, in ETHE1	2 years	Developmental regression	+	+	+	+ (Peripheral hypertonia at 4 years)	−	−	N/A	+	+	+	+	+	−	Abnormal hyperintense signals in T2W/FLAIR in bilateral putamen and caudate nuclei	Abnormal acyl-carnitine profile and metabolites on urinary analysis; easy bruising	−	Bijarnia-Mahay et al., 2016
9	c.622_624 delGAG p.E208del (paternal), c.340A>T p.I114F and c.488G>A p.R163Q (maternal) in ETHE1	3 m	Psychomotor delay	+	+	+	+	+	−	10%	+	−	+	+	+	−	Abnormal signal intensity involving the lenticular and caudate nuclei bilaterally, the brainstem and cerebellar dentate nuclei	Bilateral and symmetrical spasms of the neck, trunk and extremities with West syndrome	9 m	Papetti et al., 2015
10	c.79C>A p.Q27K and c. 554T>G p.L185R in ETHE1	15 m	Psychomotor delay	−	−	−	+	+	+	N/A	+	+	+	+	+	−	Abnormalities in the white matter, corpus callosum, and basal ganglia	Mild gastroenteritis; status epilepticus; decreased level of consciousness	−	Pigeon et al., 2009
11	c.79C>A p.Q27K and c. 554T>G p.L185R in ETHE1	6 week	Psychomotor delay	−	−	−	+	+	−	N/A	+	+	+	+	+	−	Abnormalities in the white matter, corpus callosum, and basal ganglia	Intercurrent viral illness; gastroenteritis	−	Pigeon et al., 2009
12	Homologous c.494A>G p.D165G in ETHE1	6 m	Global development delay	N/A	N/A	N/A	N/A	N/A	N/A	+	+	+	+	+	−	−	Nonspecific abnormalities	Spastic diplegia; deficiency of mitochondrial complex IV	−	Walsh et al., 2010
13	Homologous c.487C>G p.R163G in ETHE1 and c.625G>A in SCAD	2 m	Psychomotor delay	+	+	−	+	+	+	+	+	−	N/A	+	−	−	Symmetrical hyperdense lesions in T2-weighted images in the basal ganglia, the periventricular white matter, the corpus callosum and the cerebellum	Head lag at the age of 5 months; convergent strabism; no auditory interest	3 years	Zafeiriou et al., 2007
14	c.2T>A p.M1? and c.488G>A p.R163Q in ETHE1	2 years	Motor delay and language delay and regression	+	−	−	−	−	−	N/A	+	−	−	+	−	−	Lesions in the basal ganglia, bilateral lateral ventricles and corpus callosum	Hypertonia	4.25 years	Zhang et al., 2018
15	c.203T>C p.L68P and c.488G>A p.R163Q in ETHE1	7 m	Psychomotor delay and regression	+	−	−	−	−	+	N/A	+	+	+	+	+	−	Bilateral basal ganglia lesion	Hypertonia	−	Li et al., 2014

In addition, the patients in the present case, case 14 and case 15 were of Chinese origin. Although the patient in case 14 died at the age of 4.25 years, no symptoms other than chronic diarrhea, motor delay, language delay, and regression appeared. All patients of Chinese origin exhibited chronic diarrhea and developmental delay, but orthostatic acrocyanosis, hypotonia, and pyramidal signs were absent. It seems that the phenotypes in the patients of Chinese descent exhibited fewer and milder symptoms associated with vasculopathy. Since the onset-age of some patients was only a week old, we considered that different intestinal bacterium from breast milk might influence hydrogen sulfide production, and that intestinal sources of sulfur are unlikely the major factor. However, as children grown up, the exacerbation of hydrogen sulfide production and accumulation is likely associated with the increased uptake of food sources of sulfur, such as dried fruits, breads, brassica vegetables and nuts (Carbonero et al., 2012). To validate these hypotheses, more studies are required to determine the correlations between EE and dietary lifestyles, different gut bacteria or sulfur sources.

Although the pathogenesis of EE is clear, there is no well-established standard therapy for EE patients. Palliative treatments are commonly given based on clinical symptoms as follows: maintenance of hydration or caloric intake for chronic diarrhea, muscle relaxants for dystonia, antiepileptic medications for seizures, physical therapy for contractures, and L-carnitine or coenzyme Q_10_ supplements and vitamin therapies for poor energy metabolism (Gorman et al., 2016). Moreover, the combined therapy of oral metronidazole and N-acetylcysteine has been found to be an effective in EE treatment by reducing the high levels of hydrogen sulfide (Viscomi et al., 2010). Dual treatment caused marked improvement in 5 EE patients, almost without side effects as follows: body weight increased; diarrhea occurred less frequently; petechiae, acrocyanosis and seizures decreased or disappeared; hypotonia showed marked improvement; and plasma EMA significantly decreased in all 5 affected children. In addition, liver transplantation is considered as a viable therapeutic option for EE (Dionisi-Vici et al., 2016; Tam et al., 2019). Although only 3 EE patients achieved psychomotor developmental improvement and marked reversion of biochemical abnormalities after liver transplantation, their outcomes support the promising approach as a standard treatment for EE. In the present case, liver transplantation will be considered if the symptoms worsen in the future follow-ups.

In conclusion, this study described a Chinese EE patient whose disease was confirmed by WES. Two novel variants, c.586G>C (p. D196H) and c.595+1G>T, were identified and classified as “pathogenic” and “likely pathogenic.” The clinical manifestations and genetic information of all EE patients with available data were reviewed. We found that elevated EMA and C4-acylcarnitine ester are necessary conditions for an EE diagnosis and that Chinese patients have comparatively mild symptoms. Thus, for future patients with psychomotor delay, chronic diarrhea, petechiae, orthostatic acrocyanosis or hypotonia, especially those with elevated EMA and C4-acylcarnitine ester on biochemical examination, EE should be the first consideration. Using Sanger sequencing for *ETHE1* only or next-generation sequencing could provide supportive evidence to confirming the diagnosis. Our study and review are also helpful for the early and rapid diagnosis and treatment of EE patients in the future.

## Data Availability Statement

The raw data supporting the conclusions of this article will be made available by the authors, without undue reservation, to any qualified researcher.

## Ethics Statement

This study was approved by the Medical Ethics Committee of Wuhan Children’s Hospital. An informed consent form was signed by the patient’s parents for the study and the publication of this case report.

## Author Contributions

XC cared for the patient and collected the clinical data of the patient. LH interpreted the results of the genetic sequencing, reviewed the published cases, and wrote the manuscript. HY supervised the study. All authors read and approved the submitted version.

## Conflict of Interest

LH was employed by Running Gene Inc., Beijing, China.

The remaining authors declare that the research was conducted in the absence of any other commercial or financial relationships that could be construed as a potential conflict of interest.

## Footnotes

1 https://gesellinstitute.org/

2 https://db.cngb.org/

3 http://www.cbs.dtu.dk/services/NetGene2/

4 https://www.omim.org/entry/602473
